# Climatology of the United States, &c.

**Published:** 1857-11

**Authors:** 


ART. V.—*Climatology of the United States and of the Temperate Latitudes of the North American Continent, &c. &c.* By LORIN BLODGET, Author of Several Recent Reports on American Climatology; Member of the National Institute and of Various Learned Societies. Philadelphia: J. B. Lippincott & Co. London: Trübner & Co. 1857; pp. 536. Royal octavo.

CLIMATOLOGY, if not already a science, is fast advancing to the rank of one. In place of partial observations, crude conjectures, and hasty inferences, we now have a large body of carefully ascertained facts, methodically and systematically arranged on a scientific basis. That which was once regarded as a matter of philosophical curiosity, has now recognized relations with ethnology, hygiene, agriculture, and commerce. It furnishes us with formulas by which we can determine, in advance, the capability of extensive regions of the earth to be settled and cultivated by the hand of man, instead of our being obliged to wait for the results of the slow and empirical gropings, as it were, of tentative explorations and colonization. It does this by showing the temperature and other atmospherical conditions, and the physical geography of the great belts or zones in which man and his tributary animals can retain their physiological activity; and in which the great staples and secondary products for sustenance, condimental use, and clothing, can be grown, both for the inhabitants themselves, and for foreign supply.

In this volume, by Mr. Blodget, the subject of the climatology of the United States is spread before us, with its chief bearings and applications, in a spirit of painstaking and research beyond all praise. The venerable Humboldt will, we are sure, hail our author, both as a zealous disciple, and an able coadjutor, who has enlarged, filled up, and systematized the sketches which he himself made, half a century ago, as the result, in a great degree, of his travels, and the information collected in this continent. We must seek for another occasion to do justice to the present work, by exhibiting its scope, and the leading topics of which it treats, as well as the manner in which they are handled. Just now, we are unable to do more than be a mere indicator; nor can we, in the very scant space allowed us, discharge even this office in a satisfactory manner.

We cannot begin better than by giving the remainder of the title-page, the first and leading part of which heads this notice. In it the author announces its embracing a full comparison of the climatology of the North American Continent, with "the Climatology of the Temperate Latitudes of Europe and Asia, and especially in regard to Agriculture, Sanitary Investigation, and Engineering; with Isothermal and Rain Charts for each Season, the Extreme Months, and the Year; including a Summary of the Statistics of Meteorological Observations in the United States, condensed from recent Scientific and Official Publications."

A few years ago, the study of the climatology of the western or Pacific side of North America was regarded in the light of one of the curiosities of science: now it is a matter of national interest. Both in itself, and as compared with the climates east of the Rocky Mountains, it receives a full measure of the author's attention. He is the first to collect and arrange, with this view, the materials furnished by the surveys of Fremont, Emory, and Stansbury, and of those for a Pacific railroad. We can only allude to the marked contrasts in some important features of climate, between the countries on the Atlantic and Pacific, and the equally marked resemblances between the latter and the coasts of Western Europe. An interesting comparison between the arid and interior areas of the two continents is the subject of a chapter. Another is given to that of the Eastern United States with the West of Europe. To this succeeds a comparison of the basin of the Gulf of Mexico with that of the Mediterranean Sea.

"Distribution of Heat in the United States, Monthly, and for the Seasons, with Explanation of the Isothermal Charts," is the title of another division or chapter, consisting of sixty pages, which, in point of substance and information, is alike clear and comprehensive, and, taken in connection with the following, would, by some avid readers, be regarded as cheap at the price of the entire work.* The following portion is on the "Distribution of Rain, on Divisions for each Season of the Year, and in explanation of the Illustrative Charts." Another chapter is occupied with a consideration of the winds of the United States, and a comparison of them with those of Europe. This includes statements of "General Western Storms." Then comes what may be called Applied Climatology, in four successive chapters, in which are studied the climatological range of native forests, and of vegetation, and of cultivated staples of tropical or semitropical origin; also climatology of cereal grains and grasses in the United States; and general sanitary relations of the United States climate. This last topic we hope to enjoy the privilege of discussing at a more opportune time than the present. We need not lay stress on its great practical importance in order to excite the curiosity of our readers, and to induce them to consult the volume itself, in anticipation of commentaries from any other quarter. "Permanence of the Principal Conditions," and "Physical Constants," take up two more chapters. The work closes with a chapter (XIX) on the "Climate of the Northwestern Districts" of this continent, a theme which opens out a prospect of future population and agricultural products, based on scientific data, which goes beyond the imaginings of the poet.

When we consider the vast importance of the subject, the able manner in which it has been treated, and the large and liberal appliances in its

* It is no part of our business to ask, although we cannot help expressing our surprise, how this volume, made up of such paper and type, and with such an amount of tabular matter, and so many charts, can be afforded at the price of five dollars.

mechanical execution and chart illustrations, furnished by the publishers, we can have no hesitation in claiming for Mr. Blodget's volume a ready admission into every public library, whether this be one of special foundation and endowment, or of collegiate and school district, in addition to the cordial reception which it can hardly fail to receive from all students of science and readers of enlarged and philosophic views.

